# DEGRO practical guidelines: radiotherapy of breast cancer—regional nodal irradiation

**DOI:** 10.1007/s00066-026-02554-9

**Published:** 2026-06-14

**Authors:** Kai Borm, David Krug, Stefanie Corradini, Christiane Matuschek, Leonard Christopher Schmeel, Gerd Fastner, Guido Henke, Montserrat Pazos, Rene Baumann, Vratislav Strnad, Wilfried Budach, Marc D. Piroth, Nona Marciana Duma, Juliane Hörner-Rieber, Breast Cancer Expert Panel of the German Society of Radiation Oncology DEGRO

**Affiliations:** 1 Department of Radiation Oncology, TUM School of Medicine and Health, Technical University of Munich (TUM), Klinikum rechts der Isar, Munich, Germany; 2 Bavarian Center for Cancer Research (BZKF), Munich, Germany; 3 Department of Radiotherapy and Radiation Oncology, University Hospital Schleswig-Holstein, Christian Albrechts University Kiel, Kiel, Germany; 4 Department of Radiation Oncology, Universitätsklinikum Erlangen, Friedrich-Alexander-Universität Erlangen-Nürnberg, Erlangen, Germany; 5 CCC Erlangen-EMN, Comprehensive Cancer Center Erlangen-EMN (CCC ER-EMN), Erlangen, Germany; 6 Medical School and University Medical Center OWL, Klinikum Bielefeld-Mitte, Department of Radiation Oncology and Radiotherapy, Bielefeld University, Bielefeld, Germany; 7 Department of Radiation Oncology, Cologne Municipal Hospital Merheim, Hospital of the Witten/Herdecke University, Cologne, Germany; 8 Department of Radiotherapy and Radio-Oncology, University Hospital Salzburg, Landeskrankenhaus, Paracelsus Medical University, Salzburg, Austria; 9 Team Radiologie Plus, Department of Radiation Oncology, Münsterlingen, Switzerland; 10 Department of Radiation Oncology, University Hospital, LMU Munich, Munich, Germany; 11 Department of Radiation Oncology, St. Marien-Krankenhaus, Siegen, Germany; 12 Department of Radiation Oncology, University Hospital Düsseldorf, Düsseldorf, Germany; 13 Department of Radiation Oncology, HELIOS University Hospital Wuppertal, Witten/Herdecke University, Wuppertal, Germany; 14 Department of Radiation Oncology, Helios Clinics of Schwerin, University Campus of MSH Medical School Hamburg, Schwerin, Germany; 15 Department for Human Medicine, MSH Medical School Hamburg, Hamburg, Germany

**Keywords:** Regional lymph node irradiation, German Society of Radiation Oncology, Lymphatic drainage system, Individualized radiotherapy, RNI

## Abstract

**Aim:**

The aim of this work is to update the practical guidelines for adjuvant radiotherapy of the regional lymphatics in breast cancer, originally issued in 2014 by the Breast Cancer Expert Panel of the German Society of Radiation Oncology.

**Methods:**

A comprehensive literature review concerning regional nodal irradiation (RNI) was performed using the search terms “breast cancer,” “radiotherapy,” and “regional node irradiation.” Furthermore, data from recently published meta-analyses and international breast cancer society guidelines—providing new insights since 2014—served as the basis for defining evidence-based recommendations. This paper delineates the indications and dose-fractionation strategies for radiotherapy targeting the regional lymphatic pathways following breast cancer surgery.

**Results:**

The guideline was updated to reflect current evidence regarding radiotherapy of the lymphatic drainage system. The recommendations are categorized according to two primary objectives for RNI: (1) improving oncological outcomes; (2) enabling de-escalation of axillary surgery.

The update distinguishes between clinical scenarios with and without neoadjuvant chemotherapy (NACT). Specifically, it addresses the increasingly relevant topic of treatment de-escalation following pathologic complete response (pCR) after NACT in initially node-positive patients.

**Conclusion:**

The updated guidelines address current developments and ongoing challenges in RNI. They provide clinically oriented recommendations for establishing indications in both the primary (upfront) surgery setting and following neoadjuvant therapy.

**Supplementary Information:**

The online version of this article (10.1007/s00066-026-02554-9) contains supplementary material, which is available to authorized users.

## Introduction

Regional nodal irradiation is an integral component of postoperative treatment for many patients with breast cancer. Randomized evidence demonstrates its potential to improve overall survival (OS), decrease the incidence of distant metastases, and achieve durable regional control, including in patients with sentinel lymph node involvement [1]. The paradigm shift toward less invasive axillary surgery, the adoption of three-dimensional (3D) image-based and vessel-guided target volume definition, and the increasing use of neoadjuvant chemotherapy make it more difficult to interpret and apply existing evidence [2–7].

The aim of this paper is to provide a concise summary of the current evidence on regional nodal irradiation in breast cancer and to present consensus recommendations of the Expert Breast Cancer Panel of the German Society for Radiation Oncology (DEGRO) based on this evidence.

Contouring, dose constraints, treatment techniques, and detailed planning approaches are beyond the scope of the current paper. These aspects are currently under evaluation and will be addressed in a separate dedicated publication.

Historically, axillary lymph node dissection (ALND) was the standard approach to axillary management [8]. Due to the extensive nature of axillary surgery involving levels I and II, earlier randomized trials evaluating regional nodal irradiation (RNI) focused mainly on undissected lymph node regions—specifically the infraclavicular (level III), supraclavicular (level IV), and internal mammary (parasternal) lymph nodes. These studies, which included both node-positive and, with limitations (as subgroup was not separately powered in some studies), high-risk node-negative patients, demonstrated significant improvements in disease-free survival (DFS), reduced rates of distant metastases, and better regional control [1, 9–12].

To date, sentinel lymph node biopsy (SLNB) is established as the standard of care for most patients without clinically suspicious lymph node involvement. Several randomized trials consistently showed that ALND can be omitted even in the case of up to two macrometastases after SLNB if axillary irradiation is performed, which led to equivalent oncologic outcomes and significantly lower rates of lymphedema [13–15].

Accordingly, two distinct objectives of RNI can be differentiated:

1. Provide regional control *and allow for omission of ALND in patients with positive SLNB *by irradiation of the axilla *(“axillary RT”)*
2. *Improve survival in patients with high-risk breast cancer by reducing the recurrence risk *through radiotherapy (RT) of the undissected portions of the lymphatic drainage pathways and thereby eradicating potential microscopic residual disease *(“comprehensive RNI”)*

**Fig. 1:**
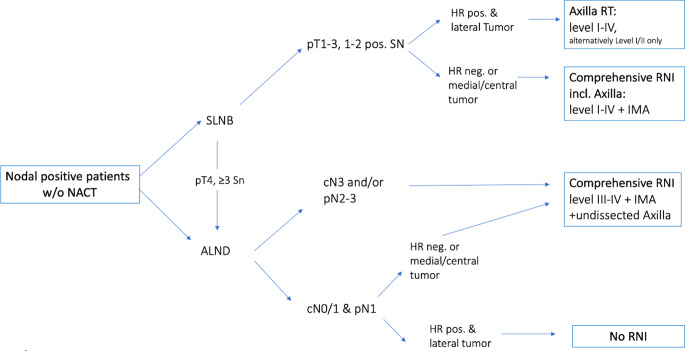
Flowchart of regional nodal irradiation (*RNI*) indication in patients with node-positive breast cancer treated without neoadjuvant chemotherapy (*NACT*). *w/o* without, *SLNB* sentinel lymph node biopsy, *SN* sentinel nodes, *HR* hormone receptor, *IMA* internal mammary artery, *RT* radiotherapy

Whereas in patients undergoing ALND only the first aspect requires evaluation, patients treated with SLNB warrant assessment of both aspects (see Fig. 1).

Due to the differential practice of axillary surgery after neoadjuvant systemic therapy and the potential impact of treatment response on the indication for RNI, the recommendations will be provided separately according to timing of surgery.

## Section 1: indication for regional nodal irradiation with up-front surgery

### Axillary irradiation in patients with positive SLNB

Several randomized trials evaluated omission of ALND in patients with cN0 status and sentinel lymph node involvement. The results demonstrated oncological equivalence if axillary RT was performed—either intentionally or incidentally—with substantially lower rates of lymphedema [13–17]. A major limitation of these trials is the heterogeneity of irradiation fields (Table 1):

- *SINODAR-One and ACOSOG Z0011, INSEMA (randomization-2)*: ALND was omitted after positive SLNB without intentional irradiation of the axilla (but with incidental radiation of levels I/II).
- *AMAROS, OTOASOR, and SENOMAC*: ALND was omitted after positive SLNB but the majority (or all) of the patients received intentional irradiation of axillary levels I–IV.
- OPTIMAL, POSNOC*: Comparison of intentional irradiation of the axilla to tangential whole-breast irradiation (*results pending).

**Table 1:** Summary of randomized trials on axillary RT

Trial	Design	Treatment arms	Axillary RT exp. arm	Inclusion criteria	*N*	Recruitment period	Median FU	Disease-free survival	Overall survival	Comment
AMAROS (EORTC 10981-22023) [14]	Multicenter RCT, phase 3 noninferiority	ALND vs. axillary RT	Levels I–IV	cN0; cT1–2, positive sentinel node (80% BCS)	1425	2001–2010	Median 6.1 y; 10‑y outcomes published	No DFS difference at 10 y (HR: 1.19, 95% CI: 0.97–1.46)	No OS difference at 10 y (HR: 1.17, 95% CI: 0.89–1.52)	Low axillary recurrence both arms; RT less lymphedema; 33% additional nodes after ALND
SENOMAC [13]	International multicenter RCT, phase 3 noninferiority	Completion ALND vs. SLNB only	Most patients recorded as receiving nodal RT covering levels I–IV	cN0, T1–T3, 1–2 macrometastatic SNs (mastectomy allowed) (64% BCS)	2540	2015–2021	Median 46.8 mo	5‑y RFS 89.7% (SLNB) vs. 88.7% (ALND); noninferior	OS not yet mature	Included mastectomy; higher nodal RT in SLNB-only; 34.5% additional nodes after ALND
OTOASOR [16]	Single-center RCT, phase 3	Completion ALND vs. RNI	Levels I–IV	cN0, cT1–2 ≤ 3 cm (80% BCS)	474	2002–2009	Mean 97 mo (8‑y follow-up)	DFS 72.1% vs. 77.4% (ns)	OS 77.9% vs. 84.8% (*p* = 0.06)	Comparable regional control; 38.5% additional nodes after ALND
SINODAR-ONE [17]	Multicenter RCT, phase 3 noninferiority	Completion ALND vs. SLNB only	Incidental dose coverage	cN0, T1–2, 1–2 macrometastatic SNs (75% BCS)	889	2015–2020	Median 34 mo (5‑y outcomes)	5‑y recurrence: 6.9% vs. 3.3% (ns)	5‑y OS ~99% in both arms	Low recurrence; 44% additional nodes after ALND
ACOSOG Z0011 [15]	Multicenter RCT, phase 3	Completion ALND vs. SLNB only (lumpectomy + WBRT)	Incidental nodal dose via tangents; ~50% received “high tangents” or incidental nodal coverage; protocol discouraged third-field RNI	T1–2, cN0, 1–2 positive SNs; (100% BCS)	891	1999–2004	Median 9.3 y (10‑y outcomes)	10‑y DFS 80.2% vs. 78.2% (HR: 0.85, ns)	10‑y OS 86.3% vs 83.6% (ns)	Incidental nodal RT in many cases; 27.3% additional nodes after ALND
OptimAL [21]	Phase III, randomized, open-label, noninferiority clinical trial	After pos. SLNB: WBRT only (INC) vs. intentional axillary nodal irradiation (INT)	Levels I–IV	cN0; BCS. low tumor burden assessed by OSNA (100% BCS)	487	2015–2021	3.7 y	INC: 93.7% vs. INT: 93.8% (difference 0.1%; noninferiority *p* = 0.075)	n. a.	Study was terminated early, which reduced statistical power. Short median FU
INSEMA (randomization 2) [22]	Randomized trial	After pos. SLNB: ALND vs. SLNB with incidental RT	Approx. 75% with incidental treatment of levels I, 20.6% received RNI in the SLNB arm	T1–T2, cN0 with 1–3 sentinel macrometastases	*N* = 386	2015–2019	5.3 y	Nonsignificant difference with HR of 1.69 for SLNB alone, iDFS rates 86.6% vs. 94.9 Locoregional recurrence was 1.1% vs. 0	94.9% vs. 96.2% (*p* = 0.66)	Early termination due to poor accrual. Trend toward worse DFS with SLND only Dropout of 25% in the ALND arm

*FU* follow-up, *HR* hazard ratio, *RCT* randomized controlled trial, *BCS* breast-conserving surgery, *ALND* axillary lymph node dissection, *SLNB* sentinel lymph node biopsy, *RT* radiotherapy, *SN* sentinel lymph nodes, *mo* months, *RFS* recurrence-free survival, *OS* overall survival, *RNI* regional nodal irradiation, *y* years, *DFS* disease-free survival, *iDFS* invasive disease-free survival, *HR* hazard ratio, *ns* nonsignificant, *n.a*. not available, *WBRT* whole-breast radiotherapy, *OSNA* one-step nucleic acid amplification

Given the substantial incidental coverage of axillary levels I–II with whole-breast irradiation with standard or high tangents in the SINODAR and Z0011 trials, a meaningful therapeutic contribution from RT to regional disease control can be assumed [16, 18]. This interpretation is supported by the fact that, across these studies, a considerable proportion of patients demonstrated additional nodal involvement on completion ALND (27–44%, see Table 1). The equivalent outcome despite relevant residual nodal disease is noteworthy and strongly suggests that RT is effective in eradicating subclinical nodal disease. The POSNOC trial will help to better understand the role of RT after positive sentinel node biopsy. The trial investigated whether additional treatment of the axilla (either RT or ALND) is necessary after positive SLNB following breast-conserving surgery or mastectomy [19]. Currently, results are still pending.

The optimal target volume for axillary RT remains unclear, as excellent regional control rates were achieved with both “incidental axillary RT” and intentional RT to levels I–IV. The SENOMAC trial can be considered the best evidence so far for omitting ALND in SLNB-positive patients due to the large sample size and the exclusion of patients with micrometastases. Notably, among comparable trials, SENOMAC also demonstrated the most comprehensive RT quality assurance, ensuring high protocol adherence and consistency across participating centers. In this trial, most patients received axillary RT to levels I–IV (level I in 55% and levels II–IV in 97%), similar to the AMAROS trial. Radiotherapy to the complete axillary lymphatic drainage (e.g., levels I–IV) is further supported by the fact that the rate of level III metastases is approximately 10% in patients with ≤ 3 positive lymph nodes in levels I–II [20]. Axillary RT confined to the caudal parts of levels I and II (approximately 5 mm below axillary vessels) can be discussed as an alternative to axillary RT levels I–IV in patients with low-risk breast cancer (e.g., one positive lymph node, ≤ T1, ECE-negative, HR-positive; lateral tumor, age > 50 years) without an indication for comprehensive RNI.

In general, the patient selection for omission of ALND after a positive SLND should be based on the inclusion criteria of the randomized trials regarding this topic. While there is clear evidence that ALND can be safely omitted in selected patients with T1–3, cN0, and ≤ 2 positive sentinel nodes after cN0 status, data are still lacking regarding the optimal management of patients with preoperatively diagnosed axillary lymph node involvement. Further evidence is required, and results from ongoing trials (AXSANA, TAXIS, ALLIANCE 11202) are awaited [23–26].

#### Recommendations

1. In patients with T1–3, cN0, and pN1a with ≤ 2 positive lymph nodes, axillary RT should be performed instead of ALND.
2. The target volume should routinely include axillary levels I–IV (additional internal mammary node [IMN] RT only if indication for comprehensive RNI; see “Axillary irradiation in patients with positive SLNB” and Fig. 1).
3. In patients with low-risk breast cancer and without an indication for comprehensive RNI, axillary RT confined to only caudal parts of levels I and II (approximately 5 mm below axillary vessels) can be considered.

### Comprehensive RNI

Irradiation of the undissected parts of the axilla, the supra-/infraclavicular lymph nodes (levels III/IV), and IMN can be considered “comprehensive RNI.”

In 2023 the Early Breast Cancer Trialists’ Collaborative Group (EBCTCG) published a meta-analysis of randomized trials focusing on regional nodal irradiation. Based on eight “modern” trials (1989–2008), a significant reduction in recurrence was detected (hazard ratio [HR]: 0.88), mainly due to a decrease in distant metastases. A significant decrease in breast cancer mortality was also observed, with a relative risk of 0.8. The absolute improvement in 15-year breast cancer mortality increased with the number of involved axillary lymph nodes: 1.6% reduction in patients without axillary lymph node metastases, 2.7% in patients with 1–3 involved nodes, and 4.5% in patients with ≥ 4 involved nodes. Additionally, patients with medial or central tumors and those with ER-negative status benefited the most [1].

Although the meta-analysis grouped all of the trials together for the effect of RNI, the included studies were very heterogeneous with respect to the targeted lymph node regions and the comparison group (Table 2). While all of the modern trials targeted the breast or chest wall in all patients, some investigated the additional effect of IMN and supraclavicular fossa (SCF) irradiation; others included the axilla, the IMN, and SCF; and some investigated the axilla and SCF. While the KROG, DBCG, and Lyon trials investigated the effect of IMN irradiation in addition to axillary/SCF irradiation, the MA.20 and EORTC 22922 trials compared SCF + IMN versus no RNI [1, 4, 9, 10, 12]. The EBCTCG subgroup analysis suggests that the reduction in breast cancer mortality depended on the inclusion of IMN. Furthermore, less than 10% of patients received RNI without the inclusion of the IMN, which further calls into question whether the benefit regarding breast cancer mortality can be extrapolated to these patients. Therefore, current German guidelines recommend inclusion of IMN in all high-risk patients receiving comprehensive RNI [27].

**Table 2:** Summary of randomized trials on comprehensive regional lymph node irradiation

Trials	Design	Treatment arms	Inclusion criteria	*N*	Recruitment period	Median FU	Disease-free survival	Overall survival	Comment
MA.20 Whelan et al. NEJM [30]	Phase 3 randomized controlled trial	WBI + Boost vs. WBI + Boost + SCF/IMN	N + or N0 high risk (T3/T2 < 10 LNs + risk Fx) (only BCS)	1832	2000–2007	8.5 y	77.0% (*p* = 0.01; HR: 0.76, 95% CI: 0.61–0.94)	81.8% vs. 82.8% (*p* = 0.38; HR: 0.91, 95% CI 0.72–1.13)	Significantly improved OS in hormone receptor-negative tumors
EORTC 22922/10925 Poortmans NEJM [31]	Phase 3 randomized controlled trial	WBI + Boost vs. WBI + Boost + SCF/IMN	N + or N0 (medial/central tumor) (75% BCS)	4004	1996–2004	10.9 y	69.1% (*p* = 0.04; HR: 0.89, 95% CI: 0.80–1.00)	77.0% vs. 78.0% (*p* = 0.06; HR: 0.87, 95% CI 0.76–1.00)	Significantly improved OS with endocrine therapy plus chemotherapy and RNI
DBCG-IMN Thorsen et al. JCO [32]	Prospective cohort study	WBI + Boost + SCF (left) vs. WBI + Boost + SCF/IMN (right)	N+ (35% BCS)	3089	2003–2007	8.9 y	n. a.	n. s.	Largest OS benefit in patients with 1–3 positive lymph nodes and medial/central tumor location, or with more than ≥ 4 involved lymph nodes
French trial Hennequin et al. UROBP [33]	Phase 3 randomized controlled trial	PMRT + SCF vs. PMRT + SCF/IMN	N+ or N0 (medial/central tumor) (only ME)	1334	1991–1997	8.6 y	49.9% vs. 53.2% (*p* = 0.35)	59.3% vs. 62.6%	Statistically underpowered and based on outdated treatment technique
KROG 08-06 Kim et al. JAMA Oncology [12]	Phase 3 randomized controlled trial	WBI/PMRT + SCF vs. WBI/PMRT + SCF/IMN	N+ (50% BCS)	735	2008–2020	8.4 y	*p* = 0.22; HR: 0.88 (95% CI: 0.57–1.14)	*p* = 0.18; HR: 0.55 (95% CI: 0.24–1.11)	Improved DFS and breast recurrence for medial/central tumor location
DBCG IMN2 [11]	Prospective cohort study	WBI + Boost + SCF (left) vs. WBI + Boost + SCF/IMN (right)	N+ (53.5% BCS)	*N* = 4541	2007–2014	13.7 y	n. a.	HR: 0.85 (95% CI: 0.76–0.94; *p* = 0.0016) for OS	Systemic treatment included taxane-based chemotherapy, aromatase inhibitors, and trastuzumab. RT was 3D-based with published QA

*FU* follow-up, *WBI* whole-breast irradiation, *SCF* supraclavicular fossa, *IMN* internal mammary nodes, *BCS* breast-conserving surgery, *y* year, *PMRT* postmastectomy radiotherapy, *HR* hazard ratio, *CI* confidence interval, *OS* overall survival, *RNI* regional nodal irradiation, *DFS* disease-free survival

**Table 3:** Summary of trials applying moderately hypofractionated regimens for RNI

Trials	Patient number	Standard arm	Experimental arm	Median FU	Primary endpoint	Statistical design	Results
HypoG-01 [68]	1265	50 in 25 Fx	40 Gy in 15 Fx	4.8 years	Lymphedema at 3 years	Noninferiority	Noninferiority for lymphedema, superiority for OS, DDFS, no diff. for LRFS and shoulder motion (not powered)
SAPHIRe [69] (Abstract)	324	50 in 25 Fx	37.5 Gy in 15 Fx	4.8 years	Perometry-assessed lymphedema within 2 years	Superiority	No statistical significantly difference between the two arms; superiority in physician-assessed lymphedema and any grade ≥ 2 toxicity for HF arm (secondary endpoint), no diff. for LRFS
DBCG Skagen 1 (Abstract) [71, 72]	2963	50 in 25 Fx	40 Gy in 15 Fx	4.12 years	Lymphedema at 3 years	Noninferiority	No significant diff. between the two arms for lymphedema, shoulder motion, breast induration as well as LRR, DR and overall death; however, increased breast cancer-specific mortality in the HF arm
Wang et al. [73]	820	50 in 25 Fx	43.5 Gy in 15 Fx	4.9 years	Locoregional recurrence	Noninferiority	Noninferiority for LRR, no difference in acute and late toxicity, less grade 3 acute skin toxicity in the HF arm
FABREC [81]	400	50 in 25 Fx	42.56 Gy in 16 Fx	3.4 years	Change in PWB domain of FACT‑B QoL at 6 months	Superiority	No significant difference in PWB change between the HF and CF arms; for pts < 45 years superior PWB at 6 months and fewer adverse effects and nausea Fewer treatment breaks and less unpaid time off from work with HF
START pilot/A/B [65]	864	50 in 25 Fx	39–42.9 Gy in 13–15 Fx	10 years	Long-term RT adverse effects	Retrospective analysis	No significant differences for patient- and physician-assessed symptoms

*OS* overall survival, *DDFS* distant disease-free survival, *LRFS* locoregional relapse-free survival, *HF* hypofractionated arm, *LRR* locoregional recurrence, *DR* distant recurrence, *PWB* physical well-being, *CF* conventionally fractionated arm, *RT* radiotherapy

While the EBCTCG meta-analysis [1] provides robust evidence for the benefits of RNI in node-positive patients, uncertainties remain regarding its efficacy in cases of limited nodal involvement (1–3 positive nodes) and regarding optimal patient selection for comprehensive RNI. While the DBCG-IMN2 study [11] suggests that comprehensive RNI—including the IMN—improves OS across all subgroups compared to RNI without IMN, the randomized trials indicate a smaller effect size in patients with only 1–3 positive lymph nodes. Consequently, German guidelines [28] recommend comprehensive RNI for these patients only when additional risk factors are present. Furthermore, there are insufficient data to determine how modern systemic therapies, such as adjuvant CDK4/6 inhibitors or HER2-targeted therapies, influence the relative benefit of RNI. Therefore, in addition to the recommendations in this section, individual clinical factors and patient preferences should be considered in the decision-making process.

The lack of an OS benefit in the SUPREMO trial [29] on postmastectomy radiotherapy (PMRT) in patients with intermediate-risk breast cancer, including those with pT1–2 N1-disease, has raised questions about the necessity of RNI in these patients. Even though 75% of patients had node-positive disease, only 10% of the patients received supraclavicular irradiation and 1% IMN irradiation. Thus, these data should not be construed as an argument not to use RNI.

It is important to note that in nearly all RNI trials, ALND was performed in all patients either as the primary procedure or subsequently following positive SLNB. In the MA.20 and EORTC trials, a median of 12 and 14 nodes, respectively, were removed [9, 10]. Hence, according to the study protocol, axillary levels I and II were not targeted in most patients. Only in the MA.20 trial did patients with < 10 removed lymph nodes or > 4 positive nodes receive additional axillary irradiation [9]. Consequently, the re-planned dose estimations of the EORTC and MA.20 trials showed low dose coverage of axillary levels I and II [34]. It should be noted that the RT treatment in these older studies was carried out with outdated techniques (2D, mixed beam, or 3D-conformal RT) and without proper image guidance. Furthermore, 3D target volume delineation and analysis of dose volume parameters were not routinely performed and targeting was instead based on anatomical landmarks.

Today, axillary surgery is often performed in a less invasive manner, limited to SLNB or targeted lymph node dissection (TAD; [35]). Even in patients undergoing ALND, there is a trend toward removing fewer lymph nodes [36]. To reproduce the excellent oncologic outcomes of the randomized trials, we recommend that, if comprehensive RNI is performed, the target volume should include the IMN, the SCF (levels III/IV), and any undissected parts of the axilla including axillary levels I and II after SLNB/TAD only.

In patients who have undergone ALND, inclusion of the dissected axillary regions in the RT target volume should be limited to cases with pathologically confirmed residual disease, other evidence of persistent nodal involvement, or extensive axillary fat tissue involvement [37]. Notably, the cranial surgical boundary for levels I/II is typically defined by the axillary vessels, which is more inferiorly than the cranial extent defined in RT contouring guidelines [4, 38]. Also, the interpectoral space (Rotter nodes) is often not fully addressed surgically, even though it represents part of the axillary lymphatic drainage. To accurately delineate undissected axillary regions, comparison of the RT planning computed tomography (CT) with preoperative imaging studies and careful review of operative and pathology reports are strongly recommended.

The benefits of comprehensive RNI have to be carefully balanced against the risk of increased treatment-related toxicity. Inevitably, RNI results in higher radiation exposure to adjacent normal tissues—particularly the heart and lungs—which, according to established dose–response models, could be associated with an elevated long-term risk of cardiac morbidity and radiation-induced secondary malignancies such as lung cancer [39]. In randomized clinical trials, however, the differences in toxicity between patients receiving RNI and those without nodal irradiation were modest. RNI was associated with a slightly increased incidence of grade 2–3 acute dermatitis and low-grade (grade 1–2) pneumonitis, whereas the overall incidence of clinically significant toxicity remained low at approximately 1% [9, 10]. In the MA.20 trial, grade 2–3 lymphedema was significantly increased with RNI (8.4% vs. 4.5%), a finding not confirmed in the EORTC 22922 trial [9, 10]. This difference may be explained by a more frequent inclusion of the dissected axilla according to protocol in the MA.20 trial. Modern series, including the DBCG IMN2 [11] cohort, show that with advanced planning techniques (3D-conformal radiotherapy, intensity-modulated radiotherapy, deep inspiration breath-hold), the risk of clinically significant late toxicity remains similar between RNI and no RNI. Even in the EBCTCG meta-analysis, there was no evidence of increased non-breast cancer mortality in trials with recruitment from 1989 onward. Especially if RNI is combined with systemic therapy, pulmonary and cardiac dose constraints and target volume coverage should be critically assessed, taking into account patient-specific factors such as cardiac risk profile and concurrent or planned systemic therapies as well as the assumed individual benefit from RNI.

#### Recommendations

- *Comprehensive RNI *aims to reduce breast cancer mortality and improve OS in patients with high-risk features.
- It should include levels III/IV, the IMN region, and any non-dissected part of the axilla.
- Comprehensive RNI is recommended for:
  - o Patients with ≥ 4 axillary lymph node metastases.
  - o Patients with internal mammary lymph node metastases.
  - o Patients with lymph node metastases in axillary levels III/IV (diagnosed in ultrasound, magnetic resonance imaging, CT, or positron emission tomography [PET]).
  - o Patients with 1–3 positive axillary lymph nodes and medial/central tumor location or negative hormone receptor status.
- Individual interdisciplinary decision-making is recommended regarding the use of RNI for patients with high-risk pN0/pN1mi disease (all risk factors: premenopausal, ER-negative, medial/central tumors, grade 3).
- Cardiac-sparing strategies, including deep inspiration breath-hold and intensity modulated radiotherapy/volumetric modulated arc therapy, should be systematically incorporated and evaluated as part of comprehensive RNI for left-sided breast cancer and should be considered for patients with right sided-breast cancer.
- Pulmonary and cardiac constraints and dose coverage should be critically assessed, taking into account patient-specific factors such as cardiac risk profile and concurrent or planned systemic therapies as well as individual risk factors for disease recurrence. Especially for IMN, a lower dose coverage may be acceptable if constraints cannot be met.

## Section 2: indication for RNI after neoadjuvant chemotherapy

The management of breast cancer patients receiving neoadjuvant chemotherapy (NACT), particularly regarding decisions on postoperative RT, has evolved considerably over the past decade. Achievement of a pathological complete response (pCR) after NACT is strongly associated with improved long-term outcomes, as shown in the CTNeoBC meta-analysis. This analysis established pCR as a robust surrogate marker for event-free survival and OS, particularly in patients with triple-negative or HER2-positive breast cancer as well as those with luminal B-like breast cancer [40]. Data from NSABP B‑18 and B‑27 (3088 patients, 335 locoregional recurrences at 10 years) further demonstrated that the risk of locoregional recurrence (LRR) after neoadjuvant therapy is influenced by age, clinical tumor characteristics, and pCR status [41]. Consistent with these findings, pooled analyses identified young age, positive nodal status, high-grade histology (G3), triple-negative subtype (TNBC), and absence of pCR as major predictors of increased LRR risk. Collectively, these results highlight pCR as a key prognostic indicator and underscore the importance of tailoring post-neoadjuvant RT based on tumor biology and treatment response [27].

### Treatment of the axilla after NACT

Traditionally, axillary staging with SLNB was conducted before initiation of NACT in patients with cN0 disease. This practice was later abandoned to allow for thorough assessment of the post-therapeutic lymph node status by SLNB. In the setting of cN+ disease at diagnosis, ALND used to be the standard of care regardless of axillary treatment response. However, multiple trials have studied the role of SLNB or targeted axillary dissection in cases of ycN0 after NACT [42].

Recently, data from the prospective AXSANA/EUBREAST‑3 registry were presented at SABCS 2025 [43]. With a median follow-up of 2 years, less invasive axillary staging (SLNB/TAD/targeted lymph node biopsy) was non-inferior to ALND regarding axillary recurrence-free interval at 3 years. Both patients with ypN0 and ypN+ were included in the cohort. Details on RT were sparse, however > 50% received axillary RT in both groups with higher rates in the SLNB/TAD/targeted lymph node biopsy group. Data from the randomized controlled ALLIANCE A011202 trial comparing ALND vs. SLNB + axillary RT in patients with SLN involvement after NACT are awaited.

Almahariq et al. [44] examined the survival impact of substituting SLNB and RNI for ALND in patients with residual ypN1 nodal disease following NACT. Using the National Cancer Data Base (NCDB), 1617 patients were analyzed, and matched comparisons revealed that SLNB plus RNI was associated with significantly inferior OS compared with ALND plus RNI (5-year OS: 71% vs. 77%, HR: 1.7, *p* < 0.001). However, in patients with only one positive sentinel node and HR-positive disease, no difference was observed. A pooled retrospective analysis of two NSABP trials on the other hand showed no difference between SLNB and ALND in patients with ypN+ if axillary RT was performed [45]. However, there were several critical limitations in the study including a small sample size (SLNB in only 31 cases) and a potential negative selection bias for ALND.

Although the current role of SLNB/TAD in patients with ypN+ remains largely unclear, it is evident that these patients have a high risk of further axillary lymph node metastases. This may be true not only in the case of SLNB but also in the case of targeted lymph node dissection with additional resection of initially suspicious lymph nodes: In the TAXIS trial preliminary data in patients with cN+/ypN+ revealed additional metastases detected by ALND in 70% of cases after targeted lymph node dissection [26, 46, 47].

The proportion of patients with residual isolated tumor cells (ITC, ypN0(i+)) following NACT in the NSABP B‑51/RTOG 1304 trial is unclear, because this information was not assessed [2]. The OPBC-05/ICARO study [48] retrospectively evaluated the role of ALND in patients with ypN0(i+) cells after NACT. They demonstrated that 10–12% of patients had additional micro- or macrometastases on ALND. Nevertheless, axillary recurrence following omission of ALND occurred in only 3.1% of patients at 3 years. However, more than three quarters (77%) of patients received adjuvant RNI.

In a subset analysis of the prospective, multicenter AXSANA cohort, completion ALND (cALND) was performed to evaluate the residual nodal tumor burden in patients with low-volume metastases (ypN0(i+) or ypN1mi) in the sentinel or target lymph node after NACT. Among 16 patients with ypN0(i+) disease, one patient (6%) was upstaged to ypN1a by cALND. Among 71 patients with ypN1mi, five (7%) were upstaged to ypN2 and one (1.5%) to ypN3. These findings indicate that additional nodal involvement may persist in a proportion of patients with minimal residual disease, supporting consideration of adjuvant axillary RT when ALND is omitted [49].

There are several ongoing trials addressing the optimal axillary treatment for patients with cN+ and ypN+ [46, 50, 51]. Until these results are available, ALND remains the standard of care with subsequent RNI including the undissected axilla (see above). For patients with ypN1mi there is debate regarding optimal therapy, and both additional ALND and TAD + axillary RT can be discussed.

While ALND is still considered standard of care for patients with ycN+ after NACT, the TAXIS trial is currently studying deescalating axillary surgery by combining TAD with removal of clinically suspicious lymph nodes during surgery instead of performing systematic ALND.

#### Recommendations after NACT

- In patients with cN+, ypN0 after SLNB/TAD, axillary RT (levels I–IV) may be considered depending on risk factors (see recommendations in “Indications for comprehensive RNI after NACT”).
- In patients with ypN0(i+) after SLNB/TAD, axillary RT (levels I–IV) may be considered taking into account individual risk factors (see recommendations in “Indications for comprehensive RNI after NACT”).
- In patients with ypN1 or ypN1mi after SLNB/TAD, axillary RT (levels I-IV) should be delivered.
- After ALND, irradiation of the dissected part of the axilla is not recommended (exceptions are axillary fat tissue invasion and residual axillary LN metastases).

### Indications for comprehensive RNI after NACT

**Fig. 2:**
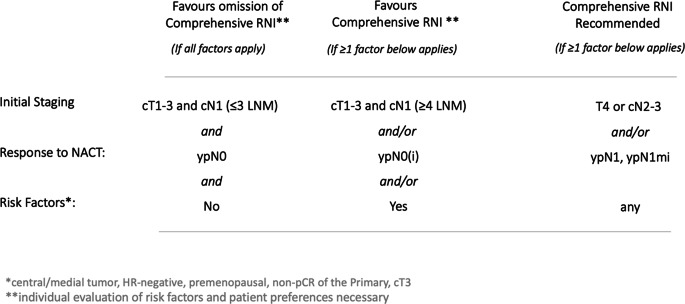
Flowchart of indications for regional nodal irradiation (*RNI*) in node-positive patients after neoadjuvant chemotherapy (*NACT*).*LNM* lymph node metastases

For all patients with clinically node-positive disease (cN+) at diagnosis, comprehensive RNI should be evaluated (Fig. 2).

Lymph node involvement should be confirmed by core needle biopsy or fine needle aspiration. Staging should include CT of the chest and abdomen with additional assessment of the neck in patients with suspicious lymph nodes in levels III/IV. Since there is currently no established consensus on how to optimally classify the degree of nodal involvement and which factors should determine the indications for RNI, assessment should include fixation of lymph nodes (as per TNM staging) as well as the number of suspicious lymph nodes on imaging. While FDG-PET/CT is not routinely performed, some data such as the MARI protocol point toward its value in assessing initial disease extent and tailoring locoregional treatment [52].

After NACT, two situations can be distinguished: patients who achieve ypN0 status and patients with residual lymph node metastases (ypN+) after NACT (Fig. 2). Upon pathological assessment, post-therapeutic changes in the assessed lymph nodes (i.e., fibrosis) might point toward initial nodal involvement. While its diagnostic validity is not completely understood, the presence of nodal fibrosis should be stated in the pathology report.

Collectively, the reviewed studies underscore the need for individualized RNI, considering the initial disease stage, extent of residual nodal involvement, tumor biology, and response to NACT.

#### Nodal complete remission (ypN0) after NACT in patients with cN+

Several studies explored the possibility of de-escalating RT in patients in ypN0 after NACT. Schlafstein et al. [53] evaluated the role of RNI in clinically node-positive (cN1) breast cancer patients who achieved ypN0 status after NACT and underwent BCS with SLNB alone. The study, using the National Cancer Database, found that adding RNI to whole-breast radiation (WBRT) did not significantly improve OS (10-year OS: 83.6% for WBRT alone vs. 79.5% for WBRT + RNI, *p* = 0.14), even after multivariable and propensity score-adjusted analyses. In the ACOSOG Z1071 secondary analysis [54], the 10-year locoregional recurrence (LRR) rate was 6.1%; achieving pCR significantly reduced LRR and improved survival, while triple-negative subtype and ypN+ increased recurrence risk. A trend toward lower LRR with RT was noted but without impact on overall or disease-specific survival. A meta-analysis of 13 studies found that adjuvant locoregional RT in patients with initially node-positive but ypN0 disease significantly reduced LRR (HR: 0.59, 95% CI: 0.42–0.81), although no benefit was observed for DFS or OS [55]. Similarly, a retrospective NCDB analysis (2010–2015, *n* = 26,009) showed that RNI was less frequently delivered in ypN0 than ypN+ patients. After breast-conserving therapy, both patients with ypN0 and ypN+ derived an OS benefit from RNI, whereas after mastectomy, no benefit was seen for ypN0 and only a trend for ypN1 disease [56].

Findings from the RAPCHEM prospective registry [57] demonstrate that risk-adapted locoregional RT resulted in low 5‑year LRR (< 4%) in patients with cT1–2 cN1 when RT was tailored based on nodal response and risk stratification. This included the omission of RNI in patients with ypN0‑1. Notably, compliance with the protocol-specified target volumes in RT was achieved in only approximately 60% of cases, while in about 30% of cases the irradiation field exceeded the intended extent; ALND was performed in 81% of patients.

The only published randomized controlled trial on this topic is NSABP B‑51/RTOG 1304, evaluating the role of RNI in patients with cT1–3 cN1 breast cancer who achieved ypN0 after NACT [58]. In this large trial of 1641 patients, RNI did not significantly improve invasive breast cancer recurrence-free interval, LRR-free interval, distant recurrence-free interval, DFS, or OS after a median follow-up of 59.5 months. There was a nonsignificant trend for improved isolated LRR-free interval, with only four recurrences in the RNI-arm versus 11 recurrences in the non-RNI arm (HR: 0.37, [0.12, 1.16], *p* = 0.088). The results are only of limited applicability for several reasons. The study suffered from a markedly lower-than-expected event rate for the primary endpoint. In comparable studies and meta-analyses such as the EBCTCG meta-analysis, improvements in oncological outcomes were demonstrated only after 10–15 years of follow-up. In addition, some subgroups were insufficiently represented, particularly patients with cT3 disease and those with residual tumor in the breast at the time of surgery. Moreover, the findings apply exclusively to patients with cN1 disease and should not be extrapolated to those with locally advanced breast cancer, high axillary tumor burden (> 4 LN initially affected), or clinical involvement of the supraclavicular or parasternal lymph nodes. Interpretation is further hampered by the fact that the trial enrolled patients both after BCS and after mastectomy. Nonetheless, the study clearly demonstrates that the post-therapeutic stage is an important factor in clinical decision-making and that patients who achieve ypN0 status after NACT may not derive substantial additional benefit from RNI. Despite the aforementioned limitations the results provide the strongest evidence to date in this setting. This reinforces prior retrospective observations, including those of Schlafstein et al. [53] and the RAPCHEM prospective registry [57], which suggested limited benefit from RNI in patients with ypN0. Importantly, the randomized design of NSABP B‑51 reduces concerns of indication bias and confounding that have affected earlier analyses, lending greater weight to the conclusion that omission of RNI may be a safe and appropriate strategy for carefully selected patients.

#### Residual nodal disease after NACT and/or advanced breast cancer (T4, N2/3)

The evidence of de-escalation of RNI after NACT focuses consistently on patients with limited initial disease (cT1–3, cN1). It is known from the EBCTCG meta-analyses that patients with advanced breast cancer have a higher risk of recurrences and a larger potential benefit of irradiation. This includes patients that are cN+ with ≥ 4 initially involved lymph node metastases, since these patients have the largest benefit from RNI with up-front surgery and a higher risk of residual undissected lymph nodes in axillary levels III/IV [20]. Given the lack of data, RNI should not be omitted in these patients even in cases of ypN 0.

Patients with ypN+ after NACT have a worse prognosis and higher risk of regional lymph node recurrences. Vega et al. [59] evaluated RNI allocation and outcomes across four NACT trials (NSABP B‑18, B‑27, B‑40, B‑41), assessing associations between RNI and LLR, DFS, and OS. They found that RNI use correlated with tumor size, ypN status, and tumor subtype. In subgroup analyses, RNI was associated with improved OS in ypN+ HER2+ disease and reduced LRR in ypN+ HR+ patients; however, in the full cohort, RNI did not significantly improve OS, DFS, distant recurrence, or LRR. Multiple retrospective analyses, including by Rusthoven et al. [60] and Stecklein et al. [61], demonstrated that RNI improves OS in patients with ypN+ following NAC. Therefore, in these patients, comprehensive RNI should be performed irrespective of initial tumor stage.

Micrometastatic lymph node involvement after NACT (ypN1mi) is associated with a higher risk of non-sentinel lymph node macrometastases and should also be treated in analogy to patients with residual macrometastases. For patients with isolated tumor cells after NACT, evidence is sparse and no clear recommendations can be made.

#### Recommendations

- In patients with cT1–2 cN0 and ypN0, RNI is generally not indicated. It may be discussed in multi-disciplinary meetings for those patients with high risk factors (all risk factors: premenopausal, ER-negative, medial/central tumors, grade 3, ypT+).
- In patients with cT1–3, N1 (≤ 3 LN), and ypN0, omission of comprehensive RNI can be discussed. In patients with additional risk factors (≥ 4 involved lymph nodes at diagnosis, ypN0(i), medial/central tumor location, HR-negative, premenopausal, ypT+), comprehensive RNI should be discussed
- In patients with cT4 and/or cN2/3 involvement (levels III/IV or IMN) comprehensive RNI is generally indicated irrespective of nodal response to NACT.
- In patients with residual lymph node disease (ypN1–3, ypN1mi) after NACT, comprehensive RNI is generally indicated.

## Section 3: fractionation schedules for regional lymph node irradiation

Over the past decade, moderately hypofractionated RT has become the standard-of-care regimen for WBRT in the absence of RNI, supported by robust evidence from several large randomized trials [62–64]. However, the applicability of hypofractionated regimens to RNI has long remained uncertain. The Ontario trial excluded nodal fields, and only a small proportion of patients in START A (14%) and START B (6%) received RNI [65].

A pooled subgroup analysis of START A/B and pilot trials, including 864 patients who received RNI, revealed no increase in arm or shoulder morbidity and no excess late toxicity with hypofractionation [65]. Additional smaller randomized studies [66, 67] confirmed equivalent toxicity and oncologic outcomes. Together with recent phase III data, these findings validate hypofractionated RNI as a safe and effective alternative to conventional schedules.

### Recent clinical trials supporting hypofractionated RNI

Recent phase III trials have provided compelling evidence supporting the safety and efficacy of moderately hypofractionated RNI, prompting a paradigm shift in clinical practice (Table 3).

The HypoG-01 UNICANCER trial compared 40 Gy in 15 fractions against 50 Gy in 25 fractions in 1265 patients with T1–3 N0–3 M0 breast cancer [68]. At a median follow-up of 4.8 years, the hypofractionated regimen was non-inferior regarding 3‑year lymphedema incidence (≈ 33% in both arms). All secondary endpoints—including overall, breast cancer-specific, locoregional-free, and distant disease-free survival— demonstrated superior outcome in the experimental arm, with comparable grade ≥ 3 toxicity, confirming the safety and efficacy for hypofractionated RNI. The Shortening Adjuvant Photon Irradiation to Reduce Edema (SAPHIRe) trial presented at ASTRO 2024 further corroborated these findings, comparing 40.05 Gy to the breast/chest wall and 37.5 Gy for RNI in 15 fractions with 50 Gy/45 Gy in 25 fractions [69]. Among 324 patients with locally advanced breast cancer, lymphedema rates were similar (29% vs. 36%), but physician-assessed lymphedema and grade ≥ 2 toxicity were significantly lower in the hypofractionated arm. Locoregional recurrence rates were low and comparable.

The DBCG Skagen 1 trial (data presented at ESTRO 2022 and 2025) randomized 2963 node-positive patients to 40 Gy in 15 fractions or 50 Gy in 25 fractions [70–72]. Lymphedema at 3 years was non-inferior. However, a higher breast cancer-specific mortality in the 40 Gy arm, with unchanged locoregional control and OS, raised questions. This signal contrasts with the HypoG-01 findings and may reflect a higher-risk population. However, a randomized controlled trial from China with 820 patients, which exclusively enrolled patients with pT3–4 and/or pN2–3 breast cancer, demonstrated non-inferior locoregional control to conventional fractionation with a regimen of 43.5 Gy in 15 fractions [73].

Additional smaller randomized studies [66, 67] confirmed equivalent tolerability and oncologic outcomes. Together with recent phase III data, these findings validate hypofractionated RNI as a safe and effective alternative to conventional schedules.

Growing data in the post-mastectomy setting also support moderately hypofractionated RNI. The Wang et al. study and the FABREC trial [73, 74] demonstrated comparable locoregional control and survival between hypofractionated and conventional regimens, with reduced severe acute skin toxicity (3% vs. 8%) in the hypofractionated arm. The FABREC trial, emphasizing patient-reported outcomes, showed similar physical well-being across regimens but improved quality of life and fewer treatment interruptions in younger patients receiving hypofractionation. The CHARM RT trial investigated PMRT after reconstruction with RNI, and preliminary results indicate no difference between hypofractionation and conventional fractionation [75].

A recent meta-analysis of 7491 patients from 27 trials [76] further supported these results, demonstrating equivalent locoregional control, OS, and toxicity across RNI and PMRT settings, with potential benefits in treatment efficiency and patient convenience.

Initial results from the FAST-Forward nodal substudy show no increase in adverse events using 5 × 5.2 Gy compared to moderate hypofractionation after 5 years. However, until the efficacy outcomes are published, the use of ultra-hypofractionation for RNI cannot be recommended outside of clinical trials [77].

### Adoption of hypofractionation in current guidelines

The ESTRO guidelines on dose and fractionation for early-stage breast cancer published in 2022 were the first European guidelines to endorse moderate hypofractionation as the preferred regimen for RNI [78]. The latest NCCN guidelines [79] now recommend both conventional and moderately hypofractionated regimens as acceptable options. The recently updated ASTRO guidelines on PMRT including RNI state moderate hypofractionation as the preferred regimen although conventional fractionation is also recommended [80].

In Germany, the AGO updated its recommendations in 2025, stating that moderate hypofractionation should be preferred to conventional fractionation for RNI [28], while the S3 guideline continues to recommend conventional regimens as an alternative to moderate hypofractionation for methodological reasons pending publication of the aforementioned phase III data.

Overall, consistent evidence from randomized trials and updated guidelines now support moderate hypofractionation (40–43.5 Gy in 15–16 fractions) as a safe, effective, and increasingly preferred standard for RNI in breast cancer [79].

### Additional nodal boost irradiation

Evidence regarding the use of a nodal boost during RNI in breast cancer remains limited, primarily derived from retrospective studies and subgroup analyses. While randomized trials have confirmed the benefit of RNI for locoregional control, none has specifically evaluated dose escalation for residual nodal disease [82–84]. Emerging data suggest that nodal boost irradiation may enhance local control in high-risk regions with acceptable toxicity, particularly in patients with residual or extensive nodal involvement [85–87]. Current NCCN guidelines and the Lucerne Toolbox recommend considering a supplemental boost for initially suspicious or biopsy-proven nodes (e.g., internal mammary or supraclavicular) that remain unresected, preferably delivered using a simultaneous integrated technique [79, 88].

#### Recommendations

- Based on the aforementioned data, moderately hypofractionated RT (typically 40–43.5 Gy in 15–16 fractions) should be the standard regimen for RNI.
- In situations where residual pathological nodes or at-risk areas cannot be surgically addressed, a supplemental nodal RT boost may be considered, aiming for an equivalent dose in 2 Gy per fraction of 60–66 Gy (e.g., SIB 48 Gy in 15 fractions).

## Supplementary Information

ESM1: Supplementary material 1

## Data Availability

All data supporting the findings of this work are included in the article.
