# Supplementary material for: DEGRO practical guidelines: radiotherapy of breast cancer—regional nodal irradiation
Source: Strahlenther Onkol. 2026 Jun 14;202(7):692–707. doi: 10.1007/s00066-026-02554-9 (PMC13290887; doi:10.1007/s00066-026-02554-9)
Supplement: Supplementary file 1 — ESM1: Supplementary material 1 [file 66_2026_2554_MOESM1_ESM.pdf]

TUM Klinikum
Rechts der Isar · MST · 81664 München

Editorial Office
Strahlentherapie und Onkologie

TUM Klinikum
Rechts der Isar

Klinik und Poliklinik für
RadioOnkologie und
Strahlentherapie

Univ.-Prof. Dr. med. S. E. Combs
Klinikdirektorin

Ismaninger Str.22, 81675 München
www.radioonkologie.mri.tum.de
www.tumklinikum.de

München, 24. April 2026

Einreichung des Manuskripts: DEGRO-Praxisleitlinien zur Strahlentherapie der Lymphabflusswege des Mammakarzinoms

Sehr geehrter Herr Professor Hecht,

im Namen der Arbeitsgruppe Mammakarzinom der Deutschen Gesellschaft für Radioonkologie (DEGRO) reiche ich hiermit das Manuskript mit dem Titel „DEGRO practical guidelines: Radiotherapy of breast cancer — regional nodal irradiation“ zur Publikation in der Strahlentherapie und Onkologie ein.

Diese Leitlinien stellen ein wesentliches Update für die klinische Praxis dar und definieren den aktuellen evidenzbasierten Standard für die regionale Lymphknotenbestrahlung beim Mammakarzinom.

Das Manuskript wurde bereits durch den DEGRO-Vorstand final geprüft und zur Publikation freigegeben.

Wir freuen uns, diese Publikation in Ihrem Journal zu veröffentlichen und danken Ihnen für die Unterstützung,

K. Borm
Kai J. Borm

Privatambulanz
Tel. +49 89 4140-4511/-4512
Fax +49 89 4140-4882
privatambulanz.radonk@mri.tum.de

Allgemeine Ambulanz
Tel. +49 89 4140-4510/-6020
Fax +49 89 4140-4880
ambulanz.radonk@mri.tum.de

Station R2a
Tel. +49 89 4140-4304
Fax +49 89 4140-4947

Direktionssekretariat
Tel. +49 89 4140-4501/-4502
Fax +49 89 4140-4477
direktion.radonk@mri.tum.de
